# Impact of *LINC00673* genetic variants on uterine cervical cancer clinicopathologic characteristics

**DOI:** 10.7150/jca.86678

**Published:** 2023-08-15

**Authors:** Yi-Hung Sun, Liang-Jou Chen, Chun-Hao Wang, Chung-Yuan Lee, Yi-Hsuan Hsiao, Shun-Fa Yang, Po-Hui Wang

**Affiliations:** 1 Institute of Medicine, Chung Shan Medical University, Taichung, Taiwan; 2 Department of Obstetrics and Gynecology, Chi-Mei Foundation Medical Center, Tainan, Taiwan; 3 School of Medicine, Kaohsiung Medical University, Kaohsiung, Taiwan; 4 Department of Medicine, College of Medicine, National Taiwan University, Taipei, Taiwan; 5 Department of Obstetrics and Gynecology, Chiayi Chang Gung Memorial Hospital Chiayi, Taiwan; 6 Department of Nursing, Chang Gung University of Science and Technology, Chiayi Campus, Chiayi, Taiwan; 7 School of Medicine, Chung Shan Medical University, Taichung, Taiwan; 8 Department of Obstetrics and Gynecology, Changhua Christian Hospital, Changhua, Taiwan; 9 Department of Medical Research, Chung Shan Medical University Hospital, Taichung, Taiwan; 10 Department of Obstetrics and Gynecology, Chung Shan Medical University Hospital, Taichung, Taiwan

**Keywords:** long intergenic noncoding RNA 673, genetic variants, cervical carcinogenesis, clincopathological parameters, 5 years survival

## Abstract

To date, no study delineates the relationships among the genetic variants of *long intergenic noncoding RNA 673* (*LINC00673*) and uterine cervical carcinogenesis as well as clinicopathological parameters and 5 years survival of cervical cancer patients in Taiwan. Therefore, the involvement of *LINC00673* polymorphisms in cervical cancer was investigated. Genotypic frequencies of three *LINC00673* polymorphisms rs6501551, rs9914618 and rs11655237 were determined in 199 patients including 115 patients with invasive cancer, 84 with precancerous lesions, and 274 control females using real-time polymerase chain reaction. It revealed that* LINC00673* polymorphisms were not found significantly related to development of cervical cancer. Cervical cancer patients with genotypes AG/GG in *LINC00673* rs6501551 had more risk to have tumor diameter larger than 4 cm as compared to those with genotype AA (*p*=0.043). Cervical cancer patients with genotype GG in rs6501551 had worse 5 years survival as compared to those with genotypes AA/AG in multivariate analysis (hazard ratio: 4.70; *p*=0.097). However, only two patients exhibiting GG were noted, and one had mortality, another had no mortality. In conclusion, larger sample size needs to verify the associations of *LINC00673* genetic variants with clinicopathological parameters and patient survival of cervical cancer for Taiwanese females.

## Introduction

If the transcribed RNA molecules are longer than 200 nucleotides and have restricted or no capability to encode amino acid sequences, they can be defined as long intergenic noncoding RNAs (lncRNAs) 1. However, there are accumulative evidences supporting that lncRNAs can affect gene presentation via many patterns, such as assembly of chromatin modifying complex, micro RNA sponges, acceleration or repression of gene presentation, increase or inhibition of DNA methylation, implication in cell proliferation, migration, invasiveness, and apoptosis, and thus are related to a variety of biological functions 2-6. Because they can exhibit oncogene or tumor suppressor roles, they are particularly associated with cancer development 7-10. The lncRNA Hox transcript antisense intergenic RNA (HOTAIR) has been investigated for oncogenic function 7, 8, 11; and long noncoding RNAs growth arrest-specific transcript 5 (GAS5) investigated for tumor suppressor function of lncRNAs as well 9, 10, 12.

Long intergenic noncoding RNA 673 (LINC00673) has been found locating on the chromosome17q25.1 13, 14. LINC00673 overexpression has been shown in non-small cell lung cancer and displays its oncogenic capacity by increasing cell growth 14. In contrast, LINC00673 exhibits a tumor suppressive function in pancreatic cancer. Arnes et al. demonstrated that pancreatic cancer cells presented more efficiency in inducing metastatic lesions if LINC00673 expression was reduced 15. It is a lncRNA among the most epithelial-enriched pancreatic ductal adenocarcinoma related (PDA) lncRNAs. LINC00673 is situated at in a recurrent, focally amplified area in PDA and is associated with a PDA-related single nucleotide polymorphism (SNP).

When there is a different allele present in the shared DNA sequence of a gene between the members of a species or paired chromosomes in a frequency more than 5% of certain population, SNP occurs 16. When the genetic variants exert a variation on the promoter region, exon or 3'-untranslated district of a gene, they may have an impact on the gene presentation, or alter the encoded amino acids, and thereafter yield the susceptibility to various diseases or cancers 16-18. A number of SNPs within lncRNA genes have been showed to exert impacts on the expression and sequence of lncRNAs, which are defined as regulatory RNAs without protein-coding capacity, and then influence the development of individual cancer and patient survival 19, 20. For example, *LINC00673* genetic variants were reported to be associated with the risk of numerous cancers including gastric cancer 21, and liver cancer 22. Moreover, in a meta-analysis study, Zhang et al. concluded that *LINC00673* rs11655237 contributed to occurrence of cancer 23. In the neuroblastoma, the previous studies reported that the *LINC00673* rs11655237 polymorphism might be associated with neuroblastoma development and susceptibility 24, 25.

Uterine cervical cancer has been reported to rank the eighth most common incidence and the eighth common mortality among female malignancy on the basis of the data from Health Promotion Administration of the Ministry of Health and Welfare as well as Annual Cancer Registry Report in Taiwan in 2016. However, until now, no research reports the relationships among *LINC00673* genetic polymorphisms, and development of cervical cancer as well as clinicopathological parameters and survival of cervical cancer patients in Taiwan. Therefore, the purposes of this study were to investigate the implications of *LINC00673* genetic polymorphism in cervical carcinogenesis, progression and 5 years survival of cervical cancer patients.

## Materials and Methods

### Enrolled female subjects

The retrospective research was conducted by investigating the associations among *LINC00673* genetic variants and the occurrence of uterine cervical cancer as well as clinicopathological parameters and 5 years survival of cervical cancer patients. A total of four hundreds and seventy-three Taiwanese females including 199 subjects suffering from uterine cervical neoplasia (115 patients having invasive cancer and 84 having precancerous lesions) and 274 normal control females were enrolled. These patients underwent standard treatment protocols at the Department of Obstetrics and Gynecology in Chung Shan Medical University Hospital in Taichung, Taiwan since February 1994 until February 2015. Two hundred and seventy-four normal females, who participated in general examination at the Outpatient Patient Department and without history of cancer of any sites, were enrolled as the control group of our study in these time. Normal cytological results were diagnosed for them and further confirmed according to the detailed colposcopic results. Chung Shan Medical University Hospital institutional review board approved the research (CSMUH No: CS18208).

### Deoxyribonucleic acid (DNA) extraction of blood samples from all subjects for the distributions of LINC00673 genetic polymorphisms

The laboratory technicians obtained the blood samples from all participants via venipuncture. Thereafter, these samples were mixed with ethylenediaminetetraacetic acid, which were previously set in Vacutainer tubes. After completing the above step, the blood specimens were immediately stored at 4℃. Then, the technicians extracted DNA from leukocytes in conforming to the description in previous publication and subsequently dissolved these extracts into pH 7.8 TE buffer 26. The DNA quality was defined after the measurement of OD260. The OD260/OD280 ratio was determined and the range of 1.8-2.0 accorded with our criteria, and was regarded as pure to prevent its cross reactivity form the existed homologous RNA in the specimens. We ultimately stored the products at -20°C and used them as the templates for the polymerase chain reaction (PCR).

### Selection of three LINC00673 genetic variants and genotyping

The selections of three *LINC00673* genetic variants, rs6501551, rs9914618 and rs11655237 were defined based on the data of International HapMap Project and previous research 27. SNP rs11655237, together with rs6501551 and rs9914618 situated at *LINC00673* with RegulomeDB score < 3 was defined as the tagSNPs from SNPinfo 27, 28. The *LINC00673* rs9914618 was selected because the *LINC00673* rs9914618 polymorphism was associated with progression of oral cancer 29 and hepatocellular carcinoma 30. The methods of gene polymorphisms determination have been described in using ABI StepOne Real-Time PCR System (Applied Biosystems, Foster City, CA, USA), and assessing the data with SDS vers. 3.0 software previously 31.

### Power and sample size calculations

Based on the hypothesis and our previous study, the frequencies of at least one mutated allele of *LINC00673* rs6501551, rs9914618 and rs11655237 were 25.5%, 35.5% and 35.3%, respectively 29. Assuming a 95% confidence interval, a p value of 0.05, a ratio of cases to healthy controls of 1:1, and at least 90% power to detect a 1.5-fold risk in gene variances of *LINC00673*, the sample sizes were about 200 case samples for gene polymorphisms of *LINC00673*.

### Statistical analysis

Analysis of variance (ANOVA) with Welch test was performed to assess the age differences among patients with cervical invasive cancer and precancerous lesions, and control females, and then the Games-Howell test was done for post hoc analysis. Hardy-Weinberg equilibrium was performed to assess the genotypic frequencies of rs6501551, rs9914618 and rs11655237 in control females [degree of freedom (d.f.) = 2].

The associations of cervical carcinogenesis with three LINC00673 genetic polymorphisms were assessed by chi-square or Fisher exact tests. Logistic or multinomial logistic regression models were performed to calculate the adjusted odds ratios (AORs) and their 95% confidence intervals (95% CIs) after age adjustment in comparing these associations. Chi-square or Fisher exact tests were also applied to relate the frequencies of three *LINC00673* SNPs rs6501551, rs9914618 and rs11655237 with several clinicopathological parameters including clinical stage, pathologic type, cell grading, cervical stromal invasion depth, tumor size, as well as parametrium invasion and vagina invasion, and pelvic lymph node metastasis.

The impacts of *LINC00673* genetic polymorphisms and clinicopathological variables on 5 years survival of patients with invasive cervical cancer were checked using Kaplan-Meier model plotting in univariate analysis. The log-rank test was applied to define the statistical significance among them. The influences of *LINC00673* genetic variants and above mentioned clinicopathological parameters on 5 years survival of these patients were assessed using Cox proportional hazard model in multivariate analysis in relation to 5 years survival intervals. The SPSS, version 25.0 and WinPepi Software, version 10.0 was applied for checking statistical significance. Hazard ratios (HRs) and their 95% confidence intervals (CIs) were also defined by the SPSS, version 25.0. *P* < 0.05 was determined to exert a significant difference.

## Results

There was significant difference for the age distribution between patients with cervical neoplasm and control females (51.2 ± 13.8 vs. 43.5 ± 10.1, *p* < 0.001). There was a significant difference among patients with invasive cancer and precancerous lesions of uterine cervix as well as control women based on the Welch test (*p* < 0.001). Using Games-Howell test as post hoc analysis, the age differences were significant between patients with cervical cancer and patients with precancerous lesions (56.1 ± 12.5 vs. 44.4 ± 12.6, *p* < 0.001) as well as between cervical cancer patients and control women (56.1 ± 12.5 vs. 43.5 ± 10.1, *p* < 0.001). But no significant difference was noted for the age distribution between patients with precancerous lesions and control females (44.4 ± 12.6 vs. 43.5 ± 10.1, *p* = 0.805).

There was no statistical difference in the distribution of LINC00673 genetic variant rs6501551 for genotypes A/A A/G and G/G between patients with cervical neoplasias and control women (*p* = 0.398). The frequencies of other LINC00673 SNPs, rs9914618 and rs11655237 exhibited no statistical significance between them (*p* = 0.915 and 0.573, respectively; Table 1). Although age was adjusted, it still did not reveal significant difference of genotypic distributions of LINC00673 SNPs rs6501551, rs9914618 and rs11655237 between patients with cervical neoplasias and control females (*p*=0.425, 0.829, and 0.543, respectively; Table 1).

Thereafter, patients with cervical neoplasias were reclassified into two subgroups i.e. patients with invasive cervical cancer and those with precancerous lesions, to investigate the relationships among the development of cervical cancer and *LINC00673* SNPs. However, it still revealed no significant association among the genotypic distributions of A/A, A/G and G/G in *LINC00673* rs6501551 and patients with cervical invasive cancer, and those with precancerous lesion as well as control females (*p* = 0.560; Table 2). Moreover, no significant associations were also found among development of cervical cancer and *LINC00673* rs9914618 and rs11655237 (*p* = 0.949 and *p* = 0.584, respectively; Table 2). Even after age adjustment, there were no relationships among the risks of cervical precancerous lesions and invasive cancer and genotypic distributions of these *LINC00673* genetic polymorphisms (Table 2).

The relationships among* LINC00673* genetic polymorphisms and clinicopathological factors of cervical cancer were further assessed. It showed that cervical cancer patients with genotypes AG/GG in *LINC00673* rs6501551 had more risk to have tumor diameter larger than 4 cm as compared to those with AA (*p* = 0.043; Table 3). Furthermore, cervical cancer patients with allele T in rs11655237 had more risk to have adenocarcinoma histologic type as compared to those with only allele C (*p* = 0.049; Table 3). However, above associations only reached marginally statistical significances.

Upon univariate analysis, distributions of *LINC00673* SNPs rs6501551, rs9914618 and rs11655237 were not found to be correlated with the 5 years survival of cervical cancer patients [*p* = 0.635, HR = 1.28 (95% CI = 0.46-3.60), AG/GG vs. AA and *p* = 0.097, HR = 4.70 (95% CI = 0.62-35.42), GG vs. AA/AG in rs6501551; *p* = 0.970, HR = 0.98 (95% CI = 0.37-2.62), GA/AA vs. GG and *p* = 0.415, HR = 2.26 (95% CI = 0.30-16.99), AA vs. GG/GA in rs9914618; *p* = 0.996, HR = 1.00 (95% CI = 0.36-2.80) CT/TT vs. CC and *p* = 0.418, HR = unavailable (u.a.), TT vs. CC/CT in rs11655237; Table 4]. Whereas, HRs with worse 5 years survival could be found in cervical patients with clinical stage ≥ II (*p* = 0.011, HR = 3.32, 95% CI = 1.25-8.86), stromal invasion > 10 mm (*p* = 0.011, HR = 3.83, 95% CI = 1.25-11.77), tumor diameter > 4cm (*p* = 0.008, HR: 3.69, 95% CI: 1.32-10.37), positive parametrium invasion (*p* = 0.032, HR = 2.70, 95% CI = 1.05-6.98) and positive lymph node metastasis (*p* < 0.001, HR = 8.95, 95% CI = 3.19-25.16; Table 4).

**Figure 1:**
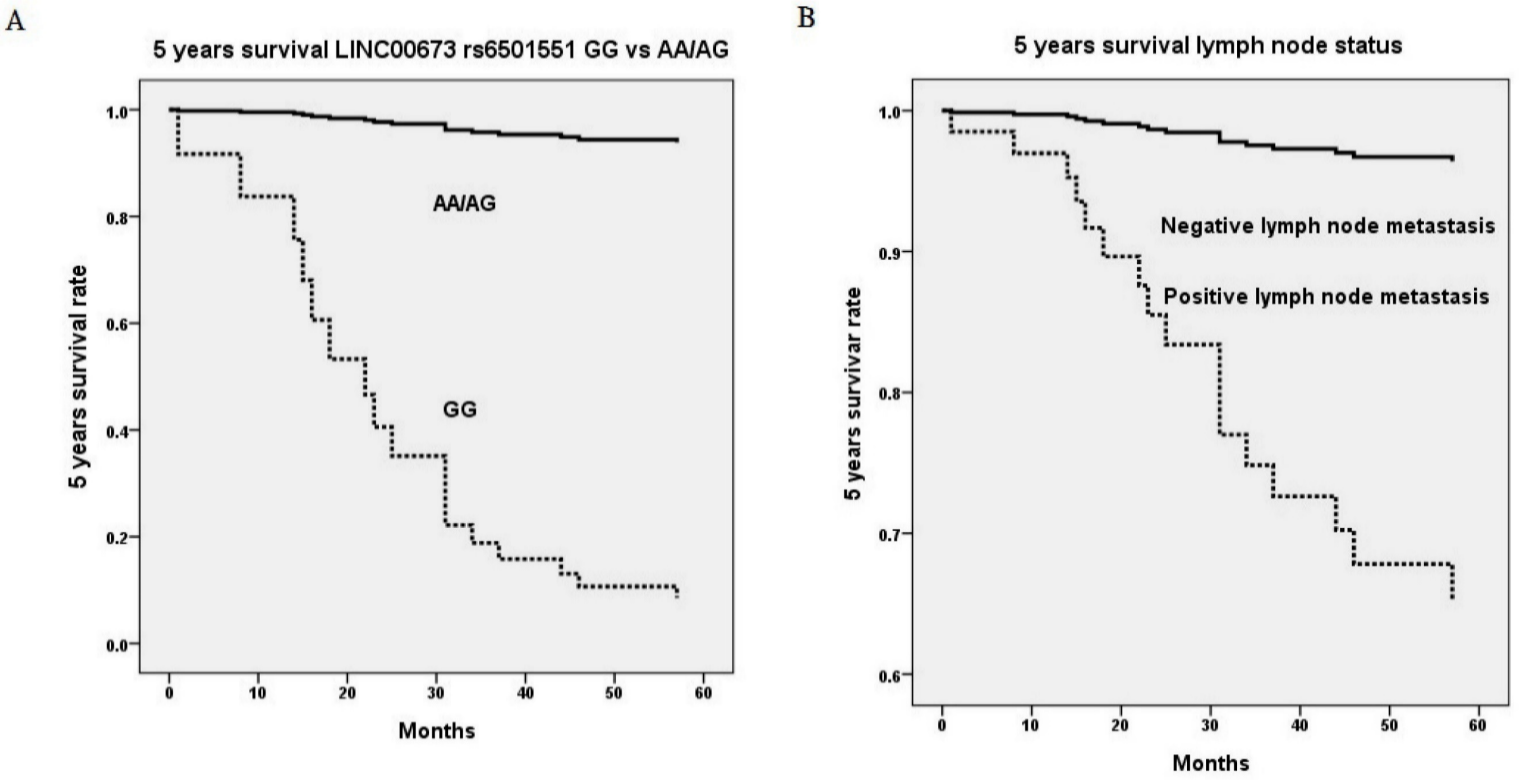
Five years survival rate based on genetic variant of long intergenic noncoding RNA 673 (LINC00673) rs6501551 (*p*=0.008, HR=38.7, 95% CI=2.55-587.95) (A) and pelvic lymph node status (*p*=0.002, HR=11.6, 95% CI=2.45-55.06) (B) using Cox proportional hazard model. HR, hazard ratio; 95% CI, 95% confidence interval.

Upon multivariate analysis including all *LINC00673* SNPs and significant univariate parameters for analysis, invasive cervical patients with genotype GG in *LINC00673* rs6501551 had worse 5 years survival as compared to those with genotypes AA/AG in Taiwanese females (*p* = 0.008, HR = 38.7, 95% CI = 2.55-587.95; Table 5, Figure 1A). Moreover, other *LINC00673* SNPs rs9914618 and rs11655237 were not related to 5 years survival. Moreover, lymph node metastasis was another factor that could predict 5 years survival of cervical cancer patients (*p* = 0.002, HR = 11.6, 95% CI = 2.45-55.06; Table 5, Figure 1B).

## Discussion

Huang et al. found that serum levels of LINC00673 were highest in patients with cervical cancer as compared to those in patients with CIN and normal controls 32. In addition, increased cell proliferation and cell cycle progression were also reported in SiHa and HeLa cervical cancer cell lines, which exhibited LINC00673 overexpression. Moreover, Shi et al. revealed that LINC00673 overexpression was noted in cervical cancer tissues and was related to poor prognosis in cervical cancer patients 33. Through negatively modulating miR-126-5p expression and further enhancing PTEN/PI3K/AKT signaling pathway, LINC00673 presents oncogenic function in uterine cervical cancer. In contrast, Wang et al. found that expression of LINC00673 was significantly reduced in cervical cancer tissues than in their normal counterparts 34.

Wang et al. further demonstrated that patients with rs11655237 allele A (T) had significantly lower LINC00673 expression. It has been reported that rs11655237 is situated on exon 4 of LINC00673 13, 35. *LINC00673* rs11655237 allele A was inferred to be related to increased risk of cervical cancer, possibly via down-regulating LINC00673 expression in cervical tissues 34. It implies that LINC00673 is a tumor suppressor in cervical cancer and the G > A (C > T) change at rs11655237 probably leads to a target site for miR-1231 binding, which reduces the function of LINC00673 34-36. In pancreatic cancer cells, Zheng et al. demonstrated when LINC00673 was bound to miR1231, it was downregulated. Then, PTPN11 was accumulated, and an oncogene IFNAR1 was upregulated, increasing the proliferation of pancreatic cancer cells 35. In addition, *LINC00673* genetic variants were also reported to be associated with the risk of a number of cancers including gastric cancer 21, and liver cancer 22. Moreover, in a meta-analysis study, Zhang et al. concluded that rs11655237 contributed to occurrence of cancer in all models in Chinese population 23. As far as our knowledge, no study explores the relationships between *LINC00673* SNPs and the development of cervical cancer in Taiwanese females. Therefore, we investigated the involvement of *LINC00673* genetic variants in carcinogenesis of uterine cervix. However in the research, three *LINC00673* genetic polymorphisms rs6501551, rs9914618 and rs11655237 could not be found to exert significantly different distributions among patients with cervical cancer and those with precancerous lesions and control females.

While correlating *LINC00673* genetic variants with clinicopathological parameters, it revealed that cervical cancer patients with genotypes AG/GG in *LINC00673* rs6501551 had more risk to exhibit tumor diameter larger than 4 cm as compared to those with AA. Moreover, cervical cancer patients with genotypes CT/TT in rs11655237 had more risk to have adenocarcinoma histologic type as compared to those with CC. But, these associations only reached marginally statistical significances. No association was found between *LINC00673* and lymph node metastasis in cervical cancer patients. As far as to our knowledge, no research investigates the involvement of* LINC00673* or its SNPs in metastasis potentials of cervical cancer. Considering in relation to clinicopathological variables, Yu et al. demonstrated that LINC00673 was related to invasion and metastasis in tongue squamous cell carcinoma (SCC) 37. Moreover, Su et al. found that genotype GA/AA in *LINC00673* rs9914618 was related to the occurrence of lymphatic spread in oral cancer as compared to GG 29. They purposed that rs9914618 is located within a CCAAT box and presents as a putative binding motif of nuclear transcription factor Y (NF-Y) 38, 39, a critical transcriptional regulator for many genes overexpression in various cancer types or CCAAT/enhancer binding proteins (C/EBPs) 40, 41, a tumor suppressor in SCC of head and neck. Furthermore, Yuan et al. suggested that the *LINC00673* rs9914618 polymorphism may be a promising HCC biomarker, especially in elderly populations 30. Above diverse findings indicated that *LINC00673* genetic variant rs9914618 probably exerted a different expression profile of cancer-associated genes mainly via impaired interactions with NF-Y or C/EBPs.

With regard to the relationships *LINC00673* SNPs with patient survival, cervical cancer patients with genotype GG in rs6501551 had worse 5 years survival as compared to those with genotypes AA/AG in multivariate analysis in Taiwan. However other *LINC00673* genetic variants rs9914618 and rs11655237 were not related to patient survival. Until now, no study reveals the relationship among *LINC00673*, its SNPs and patient survival in cervical cancer. Whereas, it has been reported that* LINC00673* was associated with poor prognosis and enhanced invasion and metastasis in tongue SCC 37. Moreover, pelvic lymph node metastasis was the only independent factor that could predict 5 years survival among various clinicopathological parameters for cervical cancer patients. As a prognosis predictor in cervical cancer, lymph node metastasis was also corroborated by previous researches 42, 43. Five years survival rate has been reported declining from 85%-90% in negative pelvic lymph node metastasis down to 30%-50% in positive lymph node with a statistical difference in patients with cervical cancer 44.

As far as our knowledge, this study may be the first research in relating the *LINC00673* polymorphisms to various clinicopathological variables and 5 years survival in cervical cancer patients in Taiwan. However, the current study has some weakness. Although cervical cancer patients with genotypes AG/GG in *LINC00673* rs6501551 had more risk to have tumor diameter larger than 4 cm as compared to those with genotype AA as well as patients with CT/TT in rs11655237 had more risk to present adenocarcinoma as compared to those with CC, these associations only had marginally statistical difference probably with a relative weak effect of specific SNPs and not significant enough to reach a definite relationship. After adjusting for *LINC00673* SNPs and various clinicopathological variables, the statistical significances might disappear. Although cervical cancer patients with genotype GG in rs6501551 had worse 5 years survival as compared to those with genotypes AA/AG in multivariate analysis, only two patients exhibiting GG were noted, and one had mortality, another had no mortality. Moreover, the study was a hospital-based cohort research and occurrence of selection bias was inevitably possible, and external validity might be limited. These problems may be resolved by enlarging the sample size in the future study. In addition, whether the *LINC00673* genetic variants affect LINC00673 expression or change the linkage to its interacting proteins or microRNAs should be delineated and need further explorations.

## Figures and Tables

**Table 1:** Genetic variant frequencies of long intergenic noncoding RNA 673 in Taiwanese females with cervical neoplasias and normal controls

Genetic variants	Normal controls (n = 274)	Cervical neoplasias^a^ (n= 199 )	ORs (95% CIs)	*p* values	AORs (95% CIs)^b^	Adjusted *p* values^b^
rs6501551						
A/A^c^	208	148	1.00	0.398	1.00	0.425
A/G	56	47	1.18 (0.76-1.83)	0.463	1.28 (0.80-2.04)	0.305
G/G	2	4	2.81 (0.51-15.55)	0.236	2.17 (0.38-12.32)	0.384
A/A^c^	208	148	1.00		1.00	
A/G & G/G	58	51	1.24 (0.80-1.90)	0.336	1.32 (0.83-2.08)	0.238
A/A & A/G^c^	264	195	1.00		1.00	
G/G	2	4	2.71 (0.49-14.93)	0.253	2.05 (0.36-11.64)	0.417
HWE values	χ^2^=0.719					
rs9914618						
G/G^c^	164	126	1.00	0.915	1.00	0.829
G/A	92	65	0.92 (0.62-1.36)	0.676	0.88 (0.58-1.33)	0.544
A/A	11	8	0.95 (0.37-2.42)	0.909	1.00 (0.37-2.69)	0.998
G/G^c^	164	126	1.00		1.00	
G/A & A/A	103	73	0.92 (0.63-1.35)	0.677	0.89 (0.60-1.33)	0.576
G/G & G/A^c^	256	191	1.00		1.00	
A/A	11	8	0.98 (0.39-2.47)	0.957	1.05 (0.40-2.78)	0.926
HWE values	χ^2^=0.181					
rs11655237						
C/C^c^	170	136	1.00	0.573	1.00	0.543
C/T	88	57	0.81 (0.54-1.21)	0.304	0.81 (0.53-1.24)	0.334
T/T	7	6	1.07 (0.35-3.26)	0.903	1.29 (0.40-4.20)	0.670
C/C^c^	170	136	1.00		1.00	
C/T & T/T	95	63	0.83 (0.56-1.23)	0.346	0.84 (0.56-1.27)	0.416
C/C & C/T^c^	258	193	1.00		1.00	
T/T	7	6	1.15 (0.38-3.46)	0.809	1.38 (0.43-4.45)	0.589
HWE values	χ^2^=1.238					

Statistical analysis: logistic regression model or chi-square or Fisher's tests. ^a^Cervical neoplasias consist of precancerous lesions and invasive cancer of the uterine cervix. ^b^The adjusted *p* values as well as adjusted odds ratios (AORs) and their 95% confident intervals (95% CIs) were calculated by logistic regression model after age adjustment. ^c^Used as a reference for comparison to assess the odds ratios of other genotypes.

**Table 2:** Genetic variant frequencies of long intergenic noncoding RNA 673 in Taiwanese females with uterine cervical invasive cancer or precancerous lesion and normal controls

Genetic variants	Normal controls (n =274 )	Precancerous lesions (n =84 )	Invasive cancer (n =115)	*p* values	AORs (95% CIs)^a^	Ad. *p* values	AORs (95% CIs)^b^	Ad. *p* values
**rs6501551**								
A/A^c^	208	61	87	0.560	1.00		1.00	
A/G	56	21	26		1.29 (0.73-2.31)	0.382	1.27 (0.70-2.30)	0.434
G/G	2	2	2		3.17 (0.43-23.09)	0.256	1.76 (0.23-13.79)	0.589
A/A^c^	208	61	87	0.555	1.00		1.00	
A/G & G/G	58	23	28		1.36 (0.78-2.39)	0.279	1.29 (0.72-2.30)	0.395
A/A & A/G^c^	264	82	113	0.383	1.00		1.00	
G/G	2	2	2		2.99 (0.41-21.71)	0.279	1.68 (0.22-13.04)	0.622
**rs9914618**								
G/G^c^	164	51	75	0.949	1.00		1.00	
G/A	92	29	36		1.00 (0.60-1.69)	0.989	0.77 (0.46-1.31)	0.342
A/A	11	4	4		1.17 (0.36-3.84)	0.794	0.85 (0.24-3.07)	0.803
G/G^c^	164	51	75	0.744	1.00		1.00	
G/A & A/A	103	33	40		1.02 (0.62-1.69)	0.933	0.78 (0.47-1.30)	0.344
G/G & G/A^c^	256	80	111	0.899	1.00		1.00	
A/A	11	4	4		1.17 (0.36-3.78)	0.793	0.93 (0.26-3.31)	0.908
**rs11655237**								
C/C^c^	170	54	82	0.584	1.00		1.00	
C/T	88	28	29		1.00 (0.59-1.68)	0.985	0.67 (0.39-1.16)	0.150
T/T	7	2	4		0.93 (0.19-4.64)	0.932	1.69 (0.41-6.99)	0.468
C/C^c^	170	54	82	0.376	1.00		1.00	
C/T & T/T	95	30	33		0.99 (0.59-1.65)	0.972	0.73 (0.43-1.23)	0.233
C/C & C/T^c^	258	82	111	0.925	1.00		1.00	
T/T	7	2	4		0.93 (0.19-4.60)	0.933	1.91 (0.47-7.84)	0.367

^a^Adjusted *p* values and adjusted odds ratios with their 95% CIs were calculated using multinomial logistic regression models after age adjustment between patients with uterine cervical precancerous lesions and control females. ^b^Adjusted *p* values and adjusted odds ratios with their 95% CIs were calculated using multinomial logistic regression models after age adjustment between patients with uterine cervical invasive cancer and control females. ^c^Used as a reference for comparison to assess the odds ratios of other genotypes. AORs, adjusted odds ratios; 95% CIs, 95% confidence intervals; Ad. *p*, adjusted *p*.

**Table 3:** Relationships between genotypic distributions of long intergenic noncoding RNA 673 and clinicopathological parameters of the patients with cervical invasive cancer

Parameters^a^	rs6501551				rs9914618				rs11655237			
	AA^b^ AG/GG		AA/AG^b^ GG		GG^b^ GA/AA		GG/GA^b^ AA		CC^b^ CT/TT		CC/CT^b^ TT	
**Clinical stage**												
stage I^b^	52	18	68	2	44	26	67	3	49	21	68	2
≥ stage II	34	10	44	0	31	13	43	1	33	11	42	2
*P* value	0.718		0.522		0.405		1.000		0.563		0.639	
**Pathologic type**												
squamous cell carcinoma^b^	77	25	100	2	67	35	100	2	76	26	99	3
adenocarcinoma	10	3	13	0	8	5	11	2	6	7	12	1
*P* value	1.000		1.000		0.765		0.062		0.049^*^		0.385	
**Cell grading**												
well (grade 1)^b^	13	3	16	0	11	5	15	1	11	5	16	0
moderate & poor (grades 2/3)	74	25	97	2	64	35	96	3	71	28	95	4
*P* value	0.758		1.000		0.749		0.455		0.774		1.000	
**Stromal invasion depth**												
≤10 mm^b^	44	13	55	2	40	17	54	3	40	17	56	1
>10 mm	37	13	50	0	33	17	49	1	37	13	47	3
*P* value	0.701		0.497		0.643		0.621		0.660		0.338	
**Tumor diameter**												
≤ 4 cm^b^	54	11	63	2	40	25	62	3	46	19	63	2
>4 cm	32	16	48	0	35	13	47	1	36	12	46	2
*P* value	0.043^*^		0.507		0.206		0.636		0.618		1.000	
**Parametrium**												
no invasion^b^	54	18	70	2	46	26	69	3	48	24	69	3
invasion	32	9	41	0	29	12	40	1	34	7	40	1
*P* value	0.715		0.534		0.459		1.000		0.063		1.000	
**Vagina**												
no invasion^b^	56	18	72	2	51	23	71	3	56	18	71	3
invasion	30	9	39	0	24	15	38	1	26	13	38	1
*P* value	0.882		0.544		0.430		1.000		0.308		1.000	
**Pelvic lymph node**												
no metastasis^b^	66	**19**	**83**	**2**	59	26	82	3	60	25	81	4
metastasis	20	**8**	**28**	**0**	16	12	27	1	22	6	28	0
*P* value	0.503		1.000		0.233		1.000		0.412		0.570	

Statistical analyses: chi-square or Fisher's exact tests. ^*^p<0.05. ^a^Clinicopathological data of some cases could not be obtained from the patients with cervical invasive cancer because of incomplete medical charts or records. ^b^As a reference.

**Table 4:** Univariate analysis of genetic variants of long intergenic noncoding RNA 673 and clinicopathological variables for 5 years survival in cervical cancer patients

	5 years survival			
Variables^a^	+	-	*P* value	HR (95% CIs)^c^
**rs6501551**				
AA^b^	71	13	0.635	1.00
AG/GG	22	5		1.28 (0.46-3.60)
AA/AG^b^	92	17	0.097	1.00
GG	1	1		4.70 (0.62-35.42)
**rs9914618**				
GG^b^	60	12	0.970	1.00
GA/AA	33	6		0.98 (0.37-2.62)
GG/GA^b^	91	17	0.415	1.00
AA	2	1		2.26 (0.30-16.99)
**rs11655237**				
CC^b^	65	13	0.996	1.00
CT/TT	28	5		1.00 (0.36-2.80)
CC/CT^b^	89	18	0.418	1.00
TT	4	0		u.a.
**Clinical stage**				
stage I^b^	60	6	0.011^*^	1.00
≥ stage II	32	12		3.32 (1.25-8.86)
**Pathologic type**				
squamous cell carcinoma^b^	85	14	0.118	1.00
adenocarcinoma	8	4		2.36 (0.78-7.18)
**Cell grading**				
well (grade 1)^b^	12	3	0.775	1.00
moderate & poor (grades 2/3)	81	15		0.84 (0.24-2.89)
**Stromal invasion depth**				
≤ 10 mm^b^	49	4	0.011^*^	1.00
> 10 mm	37	13		3.83 (1.25-11.77)
**Tmour diameter**				
≤ 4 cm^b^	56	5	0.008^*^	1.00
> 4 cm	35	13		3.69 (1.32-10.37)
**Parametrium**				
no invasion^b^	61	7	0.032^*^	1.00
invasion	30	11		2.70 (1.05-6.98)
**Vagina**				
no invasion^b^	61	9	0.115	1.00
invasion	30	9		2.07 (0.82-5.22)
**Pelvic lymph node**				
no metastasis^b.^	76	5	< 0.001^*^	1.00
metastasis	15	13		8.95 (3.19-25.16)

Statistical analyses: Kaplan-Meier curve model. ^*^p < 0.05. ^a^Clinicopathological data of some cases could not be obtained from the patients with cervical invasive cancer because of incomplete records of medical chart. ^b^As a reference. ^c^HR, hazard ratio and 95% CI, 95% confidence interval for long intergenic noncoding RNA 673 genetic polymorphisms rs6501551, rs9914618 and rs11655237 as well as clinicopathological variables, compared to their respective controls. Survival: +, survival, -, mortality.

**Table 5:** Multivariate analysis of genetic variants of long intergenic noncoding RNA 673 and clinicopathological variables for 5 years survival in cervical cancer patients

	5 years survival	
**Variables**	*P* value	HR & 95% CI^b^
**LINC00673 genetic polymorphisms**		
**rs6501551**		
AG /GG vs. AA^a^	0.537	0.64 (0.15-2.67)
GG vs. AA/AG^a^	0.008	38.7 (2.55-587.95)
**rs9914618**		
GA/AA vs. GG^a^	0.412	0.62 (0.19-1.97)
AA vs. GG/GA^a^	0.069	10.03 (0.84-120.41)
**rs11655237**		
CT/TT vs. CC^a^	0.204	2.04 (0.68-6.10)
TT vs. CC/CT^a^	0.984	u.a.
**Clinicopathological characteristics**		
**Pelvic lymph node**		
metastasis vs. no metastasis^a^	0.002^*^	11.6 (2.45-55.06)

Statistical analyses: Cox proportional hazard model. ^*^p < 0.05. ^a^As a comparison reference. ^b^HR, hazard ratio and 95% CI, 95% confidence interval for LINC00673 genetic polymorphisms rs6501551, rs9914618 and rs11655237, and clinicopathological characteristics, as compared to their respective controls. LINC00673, long intergenic noncoding RNA 673; u.a., unavailable.
